# Optimization of the Critical Speed Concept for Tactical Professionals: A Brief Review

**DOI:** 10.3390/sports9080106

**Published:** 2021-07-27

**Authors:** Nathan D. Dicks, Robert W. Pettitt

**Affiliations:** 1 Department of Nutrition, Dietetics and Exercise Science, Concordia College, Moorhead, MN 56562, USA; 2 Office of Research and Sponsored Projects, Rocky Mountain University of Health Professions, Provo, UT 84606, USA; robert.pettitt@rm.edu

**Keywords:** 3-min all-out exercise test, critical speed, HIIT, load carriage, tactical

## Abstract

Tactical professionals often depend on their physical ability and fitness to perform and complete occupational tasks to successfully provide public services or survive on the battlefield. Critical speed (CS), or maximal aerobic steady-state, is a purported measure that predicts performance, prescribes exercise, and detects training adaptions with application to tactical professionals. The CS concept has the versatility to adapt to training with load carriage as an integrated bioenergetic system approach for assessment. The aims of this review are to: (1) provide an overview of tactical populations and the CS concept; (2) describe the different methods and equipment used in CS testing; (3) review the literature on CS associated with tactical occupational tasks; and (4) demonstrate the use of CS-derived exercise prescriptions for tactical populations.

## 1. Introduction

Tactical populations include military, law enforcement, and fire and rescue personnel. Individuals in these tactical professions regularly face physically demanding tasks. These physical demands can cover a multitude of functions specific to each occupation. Military personnel during combat operations are usually required to perform periods of moderate- to high-intensity activity (e.g., the movement toward an enemy, evacuating a casualty, negotiating obstacles, finding cover), and long periods of low-intensity prolonged activity [1,2,3]. Occupational requirements for law enforcement officers could include running up and downstairs, engaging in pursuit on foot, jumping or vaulting over fences or ditches, and applying force during apprehensions [4,5,6,7]. Fire and rescue personnel requirements often include climbing stairs and ladders, hoisting hoses, performing forced entry into buildings or homes, rescuing victims, and serving as the first responding medical personnel [8,9,10,11]. These physically strenuous occupational tasks require high levels of fitness to ensure completion of the task as well as their physical safety.

Tactical professionals often carry external loads or load carriage (LC) as part of their occupation while performing the above duties, including duty gear, various equipment, weapons, body armor, and protective gear [6,10,12,13]. Such LC evokes greater demand on the aerobic and anaerobic energy systems [12]. Tactical professionals experience decreased mobility and efficiency while completing occupational tasks under LC with increased energy cost and perceptual effort [14,15]. Even though LC provides essential equipment, such a load can inhibit occupational performance (e.g., short sprints, jumping, force production) [6,16,17,18]. For this reason, training countermeasures to include aerobic exercise, resistance training, and progressive load carriage assist the tactical professional in mitigating the performance decrements as previously discussed [14,19,20,21,22,23,24]. 

The critical speed (CS) concept involves two useful variables: (1) CS serves as the mechanical measure, demarcating the heavy and severe exercise domains, associated with maximal aerobic or maximal lactate steady-state [25]; and (2) *D*′ (pronounced ‘distance prime’) represents the distance capacity of running at speeds greater than CS [26]. Exercise in the severe domain (i.e., >CS) evokes the slow component of oxygen uptake ($\dot{V}O_{2}$), meaning that the $\dot{V}O_{2}$ continues despite no change in work output or speed. Indeed, $\dot{V}O_{2}$ in the severe domain can rise and culminate with the attainment of maximum oxygen uptake ($\dot{V}O_{2}max$) at or just prior to the time of exhaustion (T_LIM_) [27]. The *D*′ is the finite energy reserve expended at speeds exceeding CS. Thus, when running at speeds above CS, *D*′ regulates the slow component’s time delay towards $\dot{V}O_{2}max$ [25]. The physiological bases for *D*′ (analogous with *W*′ from cycling) have shown to be complex with a positive correlation with the $\dot{V}O_{2}$ slow component and the loss of skeletal muscle efficiency and the development of fatigue with the accumulation of H^+^ and the depletion of phosphocreatine [25]. Running performance depends on both CS and *D*′ due to the hyperbolic relationship between running speeds, distance, and performance times [28]. Critical speed has been shown to predict completion times, load carriage performance, and prescribing interval training [19,29,30,31,32,33]. More recently, conditioning programs using the CS concept to define high-intensity interval training (HIIT) have emerged with athletic [29,34], non-athletic [30], and tactical populations [19]. With CS as a measure defining the upper boundary of the heavy-intensity domain, exercising close to CS would be described as a “high-intensity” exercise [35]. HIIT involves engaging in short bouts of exercise relative to CS with intermittent recovery sessions and is ideal for enhancing aerobic metabolism [36]. 

With the occupational task demands in tactical populations, the CS concept applies to continuous and shuttle running testing and exercise prescription to increase job task completion performance. The 3-min all-out exercise test (3MT) has been reported as an effective, valid, and reliable method to estimate CS and *D*′ for assessment and exercise prescription [37,38]. Due to the minimal amount of time and equipment, it has also been an efficient testing method. The parameters collected from the 3MT can be used to assess readiness and be used in standards for performance with tactical populations. The aims of this review are to: (1) provide an overview of tactical populations and the CS concept; (2) describe the different methods and equipment used in CS testing; (3) review the literature of CS associated with tactical occupational tasks; and (4) demonstrate the use of CS-derived exercise prescriptions for tactical populations.

## 2. Methods of Critical Speed Testing

There are various methods of testing critical speed. Considerations of time, ability, equipment, space, and facilities will often challenge practitioners. Due to shift work and training priorities, there may or may not be an abundance of time available for testing. Thus, some of the traditional methods of deriving CS and *D*′ may not be advantageous. Some organizations could also afford to purchase advanced data collection devices (e.g., GPS-enabled watches, foot pods), where others would only have minimal equipment available (e.g., cones, stopwatches). Regardless, practitioners have multiple options in administering testing. 

Traditional methods of investigating CS and *D*′ involved examining a series of exhaustive bouts at various intensities derived from a graded exercise test (GXT) or running a series of specific distances [28]. Fukuda et al. [39] used exhaustive bouts derived from the peak speed (PS) determined during a maximal GXT. Based on the participant’s performance during the maximal exercise test, these researchers prescribed four different speeds as a percentage of PS, lasting between 3 and 20 min. Similar methods were used when investigating aerobic fitness and simulated terrestrial mission task performance [40]. These researchers used four exhaustive bouts between 90 and 110% of Speed at $\dot{V}O_{2}max$, lasting between 2 and 15 min. Intensity and time to exhaustion were used in modeling the speed–time relationship to determine CS and *D*′. As previously mentioned, this testing method requires four to five sessions and can be labor intensive with larger groups and with limited time available. 

**Figure 1:**
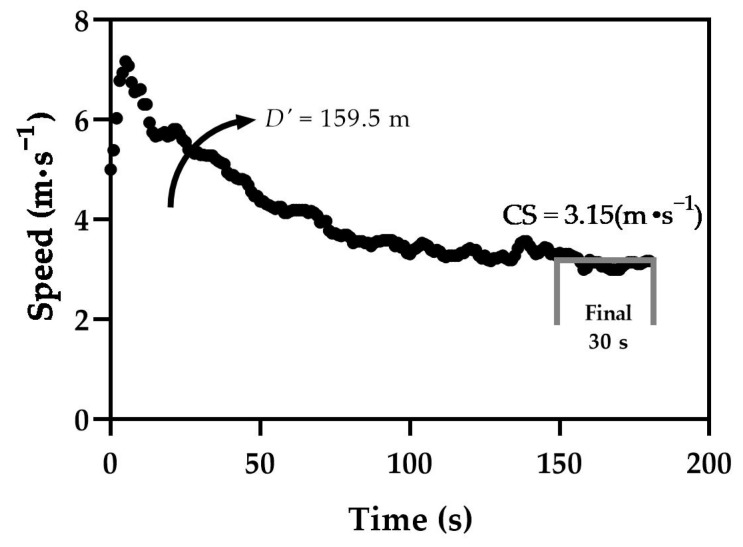
Example 3-Minute All-Out Running Test results.

The 3-min all-out exercise test (3MT) for running is used to estimate CS and *D*′ [26], which is analogous to the cycling 3MT used to assess critical power (CP) and *W*′ (pronounced work prime). The running 3MT involves the participants building up to their maximal speed progressively and maintaining that as long as possible during the entire test [26], see Figure 1. When running with all-out effort, the participant presumably expends their *D*′ within 150 s, and the CS is the estimate of the mean speed during the last 30 s of the test. The finite capacity (*D*′) to run at speeds exceeding CS is calculated from the average speed during the first 150 s (S_150s_) minus CS multiplied by the time (150 s). Some investigators have described procedures for verifying critical speed/power [38,41] with a continuous bout <CS (e.g., 20 min limit evoking a metabolic steady-state) and >CS, evoking “true” $\dot{V}O_{2}max$ and temporary exhaustion (i.e., inability to maintaining a specific speed/grade or cadence). Such verifications are certainly suitable for research investigations where CS/CP is a main dependent variable; however, research indicates such steps are not necessary for the field assessment of CS and *D*′ and HIIT exercise prescription. For use with tactical professionals, the 3MT can investigate the effects load carriage has on the integrated bioenergetic parameters [18,25] and the utility to prescribe interval training with load carriage [19,31]. 

The 3MT allows for testing a more substantial number of participants with resources commonly available for practitioners working with tactical populations. The most simplistic testing methods only require a flat indoor or outdoor track with a stopwatch and markers (i.e., cones). This method only requires the practitioner to place cones around the track at 20 m intervals. As the participants run past each cone, the practitioner would record split times using a stopwatch. The practitioner could then enter these split times into a spreadsheet where speed would be calculated using the change in displacement or distance relative to time [18,33]. The use of video can be a valid and reliable method to digitize the testing. The practitioner could set up a tripod with a digital recording device and record the testing session. With commercial editing software, the practitioner can view the video and record times at waypoints (e.g., every 20 m), calculating the speed leading up to each waypoint by dividing the distance by the change in split times. Both the stopwatch and the digitized video methods can be completed with equipment easily accessible to most. 

The global positioning system (GPS) enables sports watches to be another beneficial tool for practitioners working with athletes and tactical populations with wearable technology [26,42]. Running 3MTs can be collected outdoors on 400 m tracks or level road courses with GPS where data on time, total distance covered, and raw speed can be collected [42]. When used outdoors, the accuracy of the measurement the GPS receives is within 3 m [26] and reliable with interclass correlations coefficients of 0.96 for CS and 0.89 for *D*′ [42]. With multiple devices, data collection during testing is no more labor-intensive when a practitioner would test one or four participants at a time. 

More recently, the Stryd pod (Boulder, CO, USA) or tri-axial accelerometry footpod monitors, a wearable device attaching to the shoe, has collected data during running 3MTs [19,30]. The Stryd pod has been shown to be a reliable measure in gathering running metrics such as speed, power, ground contact time, stride length, stride rate, and distance [43]. In combination with a sports watch, 3MT testing can be completed in an indoor or outdoor environment while providing more accurate data with recorded speed data at 1 Hz [19,30]. As previously mentioned, with the use of GPS data collection can be completed on multiple participants at a time with no practitioner requirements. 

The shuttle-running 3MT could add to the capability of testing tactical professionals. The shuttle-running test was designed and validated for sports athletes such as soccer, ice hockey, rugby, and basketball [32,44]. Commonly, athletes perform acceleration, deacceleration, and direction changes at high intensity. The 3MT involves subjects running at a 25–50 m distance and switching back [32]. The length allows subjects to build up to near-maximal speed but provides several switchbacks during the test’s duration [32]. The literature on the shuttle-running 3MT did not use GPS and should be noted by practitioners, as the error with GPS (±3 m) would be compounded with the multiple changes in direction. A tactical professional’s situation will call for repetitive accelerations and decelerations at high rates with frequent changes in direction as with military maneuvers, law enforcement pursuits on foot, and firefighter tasks [6,42]. There are various methods of testing for CS and *D*′ in tactical populations. Practitioners must take into consideration time, ability, equipment, space, and facilities available. The 3MT can be carried out with minimal equipment (e.g., cones, stopwatches) or more accurate and advanced data collection devices (e.g., footpods). Practitioners have multiple options in administering testing, assessing readiness, and having the ability to prescribe HIIT with tactical populations. 

## 3. Association of Critical Speed in Occupational Tasks

Critical speed, a measure to evaluate aerobic capacity or aerobic fitness [25] with tactical populations, has shown to be a predictor associated with occupational task completion [9,22,45]. Aerobic capacity in tactical populations has been assessed through GXT [9,45] and the 20 m progressive shuttle run test [22]. The use of 3MT or multiple exhaustive bouts to model CS and *D*′ from the speed–time relationship has also been applied to various tactical populations [18,19,33,39,42]. 

Commonly, tactical populations will assess aerobic fitness through running distance tests (e.g., 1.5–2 m or 2.4–3.2 km) [39]. More specifically, the Army Physical Fitness Test is a 3.2 km or 2 m run [46]. Fukuda et al. [39] recruited seventy-eight recreationally active (1–5 h/wk aerobic exercise, resistance training, and/or recreational sports) college-aged men (n = 39) and women (n = 39). The authors suggested an alternative with the CS concept over the 3.2 km run, with the test described using multiple exhaustive bouts to model the speed–time relationship. The authors deemed it valuable in the Special Forces evaluations or Fit for Duty applications [39]. The authors concluded that the CS test offers comparable or a more accurate prediction of $\dot{V}O_{2}max$ and a way of assessing aerobic or anaerobic training needs. 

As previously mentioned, due to the additional parameters needed to collect CS and *D*′, it is ideal for assessing smaller units over large-scale implementation in the military. The logistics of testing large groups of soldiers should be factored into a limited availability of time [39]. Special Forces units present ample opportunities to benefit from the assessment of the 3MT. Hoffman et al. examined the relationship between CS and anaerobic distance capacity, or *D*′, to combat-specific tasks (CST) in an Israeli Special Forces (SF) unit [42]. The SF soldiers completed a 3MT, and the CST consisted of a 2.5 km run, 50 m casualty carry with a 60 kg manikin, and a 30 m repeated sprint with “rush shooting” (RPTDS) with their combat gear (combat vest with ammunition and helmet). The RPTDS required the soldiers to perform a 15 m sprint to and from a firing line, and shoot three rounds at a target 30 m down range (i.e., a total of five 30 m sprints and 15 shots). Researchers measured the time to complete sprints, not shooting. The authors concluded that CS was highly predictive of total performance, with partial correlations during the 2.5 km run (r = −0.55) and the RPTDS with BMI controlled (r = −0.70).

Load carriage is an inherent part of tactical professionals’ occupations and will usually limit effectiveness [6,16,17,18]. Solomonson et al. [18] recruited a sample of 14 males from various occupational backgrounds (e.g., military, law enforcement, and firefighter) to examine the effect LC has on CS. The participants completed two 3MTs on separate trials: one trial running all-out wearing an 18.86 kg weighted vest and a second unloaded trial. The loaded trial was associated with 15 to 25% of the participant’s body mass. Individuals with higher absolute body mass had a lower % of body mass carried by the fixed load (18.86 kg) where the opposite was true with those with a lower absolute body mass. The authors reported that LC evoked a decline of 0.66 ± 0.24 m·s^−1^ in CS during the 3MT and observed a linear trend between the load and the ∆CS. From the trend, a predictive equation can be used to quantify an adjusted CS using

Adjusted CS = original CS = (−0.0638 × % Load) + 0.0692,(1)

where the original CS is derived from the unloaded 3MT. The authors concluded that the LC performance was highly dependent on CS. The practical application of this research was that an unloaded 3MT may be used to predict time limits for running with different load carriages ranging from 15–25% of BM and could be used to prescribe HIIT with loads ranging from 15–25% of BM.

The predictive equation from Solomonson et al. [18] was further evaluated and validated using college Army ROTC cadets [33]. These authors examined the influence of external load carriage on short/middle distance sprinting performance by validating estimated decreases in CS from loaded sprints of 800 m and 1000 m with 20% and 15% of the participant’s body mass. The typical error of predicting actual times for the 800 m and 1000 m loaded sprints were 5.6 and 10.1 s. In conclusion, when using shorter intervals (<800 m or <180 s) for HIIT, the slight overestimation with the regression equation would be moot. These shorter intervals would be advantageous to yield increases in CS with a minimal negative impact on *D*′ [33].

There have been several studies that have used the CS model applied to tactical populations. Findings from these studies have observed CS to be a measure associated with occupational task completion performance, assessing an integrated bioenergetic system approach for training, and load carriage performance. The 3MT may have some potential utility as a criterion for unit selection and as a further predictor of combat readiness. 

## 4. Critical Speed-Derived Exercise Prescriptions for Tactical Populations 

The CS concept can derive exercise prescriptions for athletic and healthy adult populations [29,30,34]. Of the programs found in the literature, two of the studies used running as the training mode [29,30], and one used swimming [34]. Two of the programs followed four weeks [29,34] and the other followed six weeks [30]. All of the programs involved two sessions per week. All of the programs evoked significant improvements in CS. As the prescription of HIIT utilizing CS and *D*′ will provide a practitioner with intervals in the severe exercise domain, the intervals can be prescribed by a percentage of *D*′ either through time or duration expressed as follows:

St = [(*D*′ × Depletion of *D*′ Percentage)/tLIM] + CS, (2)

where St is the interval speed, and tLIM would be the interval time in seconds, or by distance

**Figure 2:**
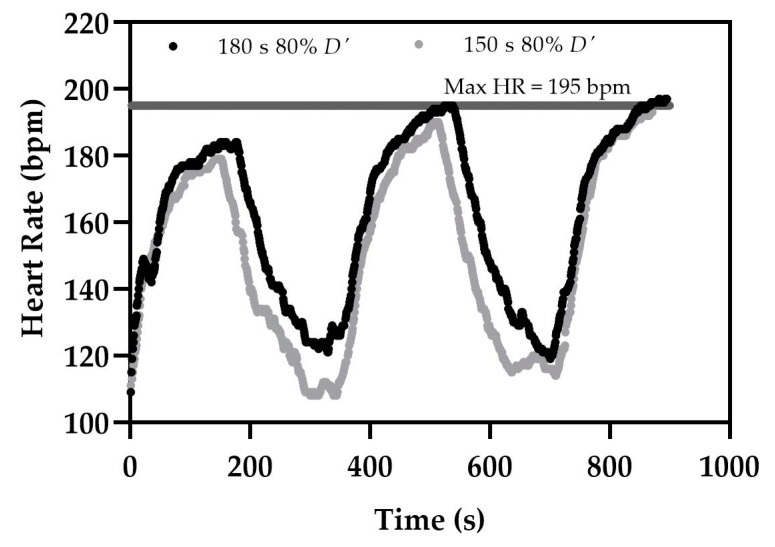
Example heart rate responses from 180 s intervals depleting 80% *D*′ and 150 s intervals depleting 80% *D*′.

Int_t_ = [*D* − (*D*′ × Depletion of *D*′ Percentage)]/CS, (3)

where Int_t_ is, the interval time and *D* is the distance. Researchers reported that when the participants are depleting 60% of *D*′, they can complete four intervals, and when depleting 80%, they can complete three intervals [29,34], see Figure 2. Due to these intervals prescribed in the severe domain of exercise, there is no steady-state even with the adjustment of speed and intensity, and presumably, participants obtaining $\dot{V}O_{2}max$.

The prescription of HIIT relative to $\dot{V}O_{2}max$ is widespread, however, there are many problems with such an approach. Typically, investigators will conduct a GXT and assign the highest power output or speed reached at the end of the test as the power/speed evoking $\dot{V}O_{2}max$. Technically, the intensity achieved at the end of a GXT is supported by aerobic and anaerobic energy systems [47]. Yet, when a new stage is introduced, there is a time lag in the $\dot{V}O_{2}$ kinetics [48] and therefore interpolation methods should be implemented [49]. Assuming investigators consider the issue above, for prescribing running intervals on the track, one must still convert differences between treadmill and overground running speeds and adjust, when necessary, converting the use of increased treadmill gradient in the GXT protocol to sufficiently evoke $\dot{V}O_{2}max$ [50]. Such adjustments can be made [51] but add complexity to the prescription. Finally, due to the nature of the CS/CP concept, a wide range of intensities can evoke $\dot{V}O_{2}max$. In other words, a variety of GXT slopes can evoke the same $\dot{V}O_{2}max$ with varying differences in peak speed/power, meaning the percentage of prescribing HIIT based on $\dot{V}O_{2}max$ is protocol-dependent (for a clear example of the problem, see Figure 7 in the recent review by Jamnick et al. [52]). 

The irony of the CS concept, which can be assessed in the field using the running 3MT on the track [26] and with tactical load [18,33], is that the technique may exceed the utility of treadmill testing in a laboratory. There is simply no error/variance introduced by using interpolation, converting treadmill to overground speeds, or any need for expensive equipment. Moreover, as opposed to the speed evoking $\dot{V}O_{2}max$ which can be varied or protocol-dependent (see above paragraph), CS is highly reliable [37]. As a result, HIIT prescribed using the CS/CP concept are robust to different interval durations and intensities, evoking consistent and predictable metabolic responses [29,53,54]. Exercise intensities based on percentages of $\dot{V}O_{2}max$ are dogma but completely uninformed on different exercise intensities based on physiological cut-points [52,55]. 

With application to tactical populations, completing HIIT can increase aerobic capacity, and CS positively associates with technical and combat-specific performance measures [18,33,39,42]. Using estimates of CS and *D*′ from the 3MT, practitioners can also prescribe HIIT for tactical populations with LC. Progressive LC is a fundamental part of exercise training programs to improve LC performance. Including LC in the exercise training adds to the specificity with adaptations to both musculoskeletal and aerobic fitness. Ideally, the research suggests regular load carriage activities two to four times a month to increase LC performance [12]. Furthermore, a regression equation identifies the relationship between the load carriage percent of body mass (15–25%) and decreases in CS [18]. After participants complete an unloaded running 3MT, practitioners can use the regression equation (Equation (1)) to prescribe interval training with an assigned load carriage amount (15–25% body mass). 

Recent research investigated the impact of using the CS concept to prescribe HIIT regimens to enhance CS and load carriage performance [19]. Twenty ROTC cadets were randomized into two training groups (LC HIIT and HIIT) completing a four-week training period with two sessions per week. The LC HIIT group’s cadets completed one of their weekly sessions with LC, where their speed for the interval was calculated from the regression equation [18]. The LC HIIT progressively increased from 15, 18, 21, and 24% of body mass for LC during each of the four weeks. The other group completed two HIIT sessions a week (without LC). After the four-week training period, both groups experienced improvements in CS, the HIIT group improved 4.8%∆, and the LC HIIT improved 1.6%∆. The LC task was a time trial completion of 3200 m with a 21 kg weight vest on an indoor track. The LC HIIT group saw moderate improvement in the 3200 m load carriage task with a −9.8%∆ where the HIIT group only saw a −5.4%∆.

The use of LC in HIIT prescriptions has shown to be another independent factor in prescribing exercise. In the study by Dicks et al. [19], the LC HIIT group underwent progressive increases in LC for the interval prescription from week to week, which yielded a small effect on increasing CS and decreasing *D*′. Yet, the improvement of the LC HIIT group on the load carriage task of 3200 m produced a large effect (*d* = 0.64). Future inquiry with load carriage and HIIT prescribing should focus on manipulating the % of body mass used and the training time better to understand the long-term effects on CS, *D*′, and load carriage performances. 

## 5. Conclusions

In summary, tactical populations may need to perform physically strenuous occupational tasks that require high levels of aerobic fitness. The use of the 3MT has shown to be advantageous for tactical populations in predicting CS and *D*′ as the test only needs one session and can be completed with a substantial number of participants with resources commonly available (e.g., stopwatch, cones, sports watches). Critical speed serves as a measure to evaluate aerobic fitness and can also serve as a predictor of occupational task performances to include load carriage. 

Exercise training programs involving CS-derived interval training would help in decreasing the effects load carriage has on running economy and speed, thus improving the ability of combat soldiers to survive [19,56]. The use of the CS concept to prescribe HIIT provides a method to keep run volume down to meditate injuries from overuse and increase running and load carriage performances [19]. Furthermore, the results from the 3MT can provide parameters for practitioners to prescribe individualized HIIT for each tactical professional.
